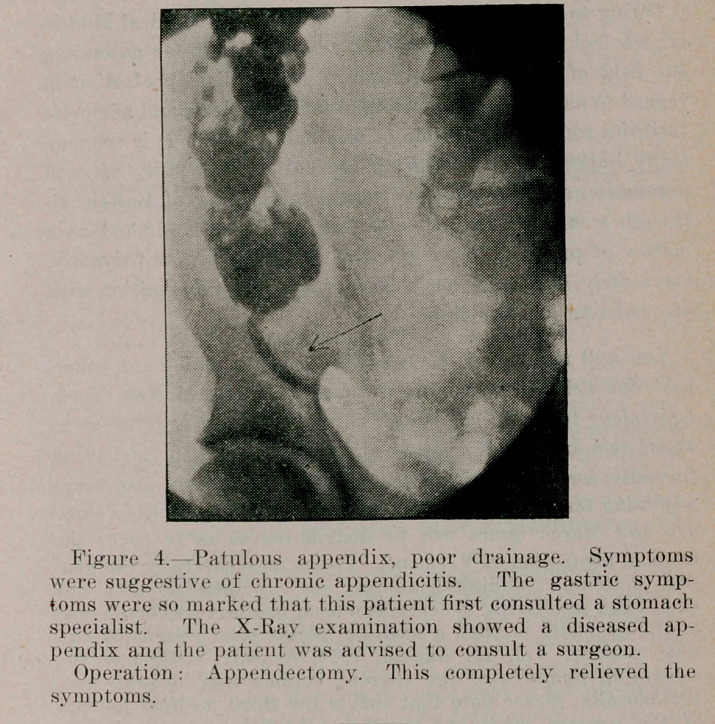# X-Ray Diagnosis—Patulous Appendix

**Published:** 1915-02

**Authors:** 


**X-Ray Diagnosis—Patulous Appendix.** J. W. Squires, Charlotte, N. C., "Charlotte Med. Jour.," January, 1915. Cuts by courtesy of editor and author. (Compare with description of James A. MacLeod and Frederick B. Bowman in our issue of April, 1914.)

Figure 1.—Patulous appendix, drainage poor. Gastric symptoms were predominant in this case. There was, however, a history of recurring attacks of abdominal pain during the past three years.

Operation: Appendectomy. This completely relieved the symptoms.

Figure 2.—Patulous appendix, drainage good. No history of appendicitis; gastric symptoms predominant. The stomach radiographically, showed evidence of a duodenal ulcer but a diagnosis of a reflex condition of the stomach probably from a chronic appendix was made. This, we think, is correct but not confirmed as the operation has been postponed.

Figure 3.—Patulous appendix, drainage poor. Stasis in terminal ileum. History of chronic indigestion. No conclusive evidence of appendiceal inflammation. Gastric disturbances predominant. No operation but the radiographic findings show beyond doubt a chronic appendix with considerable reflex gastric symptoms.

Figure 4.—Patulous appendix, poor drainage. Symptoms were suggestive of chronic appendicitis. The gastric symptoms were so marked that this patient first consulted a stomach specialist. The X-Ray examination showed a diseased appendix and the patient was advised to consult a surgeon.
Operation: Appendectomy. This completely relieved the symptoms.

## Figures and Tables

**Figure 1. f1:**
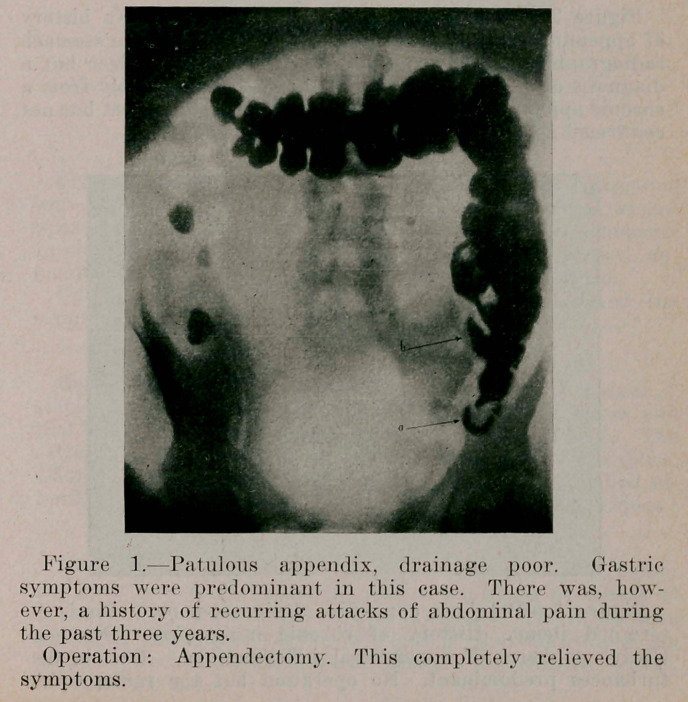


**Figure 2. f2:**
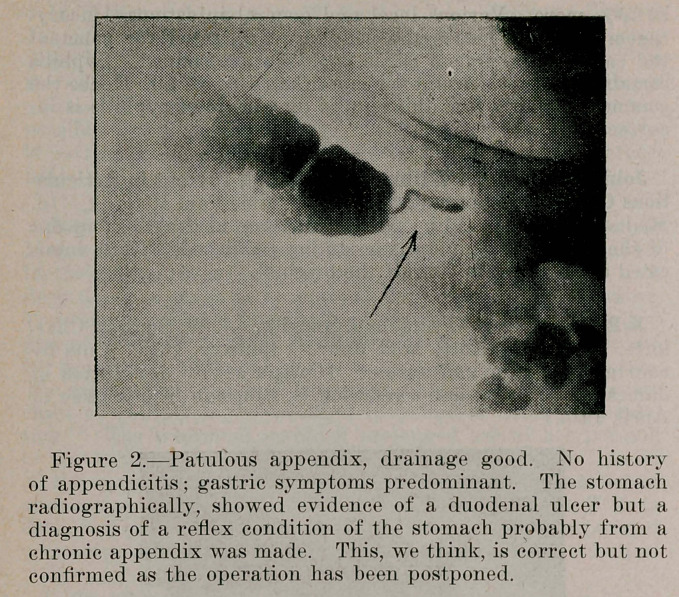


**Figure 3. f3:**
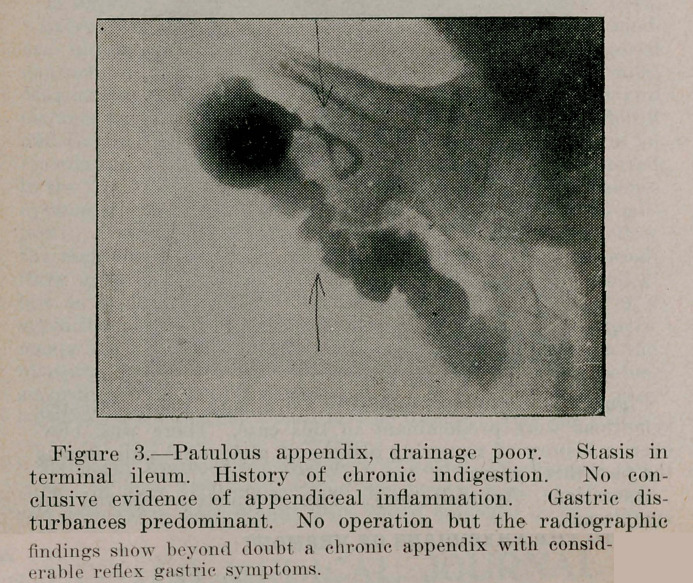


**Figure 4. f4:**